# Transcriptome profiling of resistance response to *Meloidogyne chitwoodi* introgressed from wild species *Solanum bulbocastanum* into cultivated potato

**DOI:** 10.1186/s12864-019-6257-1

**Published:** 2019-11-28

**Authors:** Sapinder Bali, Kelly Vining, Cynthia Gleason, Hassan Majtahedi, Charles R. Brown, Vidyasagar Sathuvalli

**Affiliations:** 1 Department of Plant Pathology, Washington State University, Pullman, Washington 99164 USA; 2 Department of Horticulture, Oregon State University, Corvallis, Oregon 97330 USA; 3 Retired from United States Department of Agriculture, Prosser, Washington 99350 USA; 4 Hermiston Agricultural Research and Extension Center, Oregon State University, Hermiston, Oregon 97838 USA

**Keywords:** *Solanum tuberosum*, RNAseq, Root-knot nematode, Hypersensitive response, Salicylic acid, Polyamines, Suberin

## Abstract

**Background:**

*Meloidogyne chitwoodi* commonly known as Columbia root-knot nematode or CRKN is one of the most devastating pests of potato in the Pacific Northwest of the United States of America. In addition to the roots, it infects potato tubers causing internal as well as external defects, thereby reducing the market value of the crop. Commercial potato varieties with CRKN resistance are currently unavailable. Race specific resistance to CRKN has been introgressed from the wild, diploid potato species *Solanum bulbocastanum* into the tetraploid advanced selection PA99N82–4 but there is limited knowledge about the nature of its resistance mechanism. In the present study, we performed histological and differential gene expression profiling to understand the mode of action of introgressed CRKN resistance in PA99N82–4 in comparison to the CRKN susceptible variety Russet Burbank.

**Results:**

Histological studies revealed that the nematode juveniles successfully infect both resistant and susceptible root tissue by 48 h post inoculation, but the host resistance response restricts nematode feeding site formation in PA99N82–4. Differential gene expression analysis shows that 1268, 1261, 1102 and 2753 genes were up-regulated in PA99N82–4 at 48 h, 7 days, 14 days and 21 days post inoculation respectively, of which 61 genes were common across all the time points. These genes mapped to plant-pathogen interaction, plant hormonal signaling, antioxidant activity and cell wall re-enforcement pathways annotated for potato.

**Conclusion:**

The introgressed nematode resistance in PA99N82–4 is in the form of both pattern-triggered immune response and effector-triggered immune response, which is mediated by accumulation of reactive oxygen species and hypersensitive response (HR). Salicylic acid is playing a major role in the HR. Polyamines and suberin (a component of the Casperian strip in roots) also play an important role in mediating the resistance response. The present study provides the first ever comprehensive insights into transcriptional changes among *M. chitwoodi* resistant and susceptible potato genotypes after nematode inoculation. The knowledge generated in the present study has implications in breeding for CRKN resistance in potato.

## Background

*Meloidogyne chitwoodi* Golden, O’Bannon, Santo & Finley commonly known as the Columbia root-knot nematode (CRKN) is one of the most severe pests of potato in the Pacific Northwest (PNW). This nematode was first reported in several areas in the PNW in 1977 [1] and populations flourish in the sandy soils of this major potato production region of the United States. In the PNW region, *M. chitwoodi* exists as two different races (race 1 and race 2), which can be differentiated based on their host specificity [2, 3]. It has a very short life cycle (~ 23 days) so, the nematode populations multiply rapidly under favorable conditions. The second stage juvenile (J2), the only infective stage, enters the potato root and developing tuber tissue through the epidermis by piercing the cell wall with its stylet and migrating to the root cortex [4]. In the root cortex, it establishes itself and induces the procambial cells to become giant multinucleate cells, a source of nutrients for the growing nematode [5, 6]. The cells surrounding the nematode and the giant cells divide, causing the formation of galls in both roots as well as tubers. In potato, CRKN infection does not cause noticeable root galling however, infected tubers show external galls as well as internal blemishes, which render the tubers unmarketable [7]. Presently, soil fumigation with soil sterilizing chemicals is the most effective treatment for controlling CRKN but these chemicals are a major concern because of their high costs and harmful environmental effects [8]. Host genetic resistance is viewed as a more sustainable approach to control CRKN, but to date, there has been no commercial potato variety available with genetic resistance to CRKN.

*Meloidogyne chitwoodi,* like other *Meloidogyne* species, manipulates the host’s cellular machinery to establish a continuous supply of nutrients from the living host cells. The infection cycle starts with the secretion of nematode “effector” proteins synthesized in the nematode esophageal glands, hypodermis and amphids into the host cells [9], which initiates feeding site formation. These effectors, when secreted into a resistant plant, activate a cascade of events leading to a plant immune response, which could be classified either as PAMP-triggered immunity (PTI) or as effector-triggered immunity (ETI) [10]. PTI is considered to be the first line of defense response in plants, and is usually triggered by extracellular receptor proteins such as receptor like kinases (RLKs) and receptor like proteins (RLPs) [11]. Specific intracellular proteins that recognize the pathogen effectors generate the second line of defense response or ETI. These intracellular proteins are usually referred to as disease resistance genes (R-genes) [12]. Direct or indirect recognition of pathogen proteins by the R-gene(s) triggers ETI, which often results in a hypersensitive response (HR) causing tissue lesions and (or) programmed cell death in the host. The induction of HR as part of the nematode-resistance response is similar to the R-gene mediated resistance response against root-knot nematodes in tomato (*Mi*) [13], coffee (*Mex1*) [14] *and Prunus* spp. (*Ma1*) [15]. Thus, host-specific resistance to root-knot nematodes typically involves an HR, which blocks successful feeding site formation or expansion of the feeding sites [16].

The advent of highly sensitive, effective and inexpensive direct mRNA sequencing technology and the availability of reference genomes for most of the major crops has made it possible to study the differential gene expression between compatible and incompatible host-nematode interactions. Transcriptome profiling has been used as an effective tool to study the resistance and susceptible response to *M. incognita* in alfalfa [17], tobacco [18, 19], tomato [20] and sweet potato [21]. To date, most host-nematode transcriptomic studies have been done with the tropical root-knot nematode *M. incognita* in various host crops such as alfalfa, tobacco, tomato and sweet potato [17–21]. Understanding of the resistance response in potato challenged by *M. chitwoodi* is lacking.

Screening of wild potato species identified *M. chitwoodi* resistance in *Solanum bulbocastanum*, *S. hougasii, S. stenophyllidium* and *S. fendleri* [22–24]. The resistance identified from clone 22 of diploid *S. bulbocastanum* (SB22) was hybridized with cultivated tetraploid *S. tuberosum* using protoplast fusion. The somatic hybrid obtained by fusion was subsequently backcrossed five times with various tetraploid *S. tuberosum* genotypes resulting in nematode resistant advanced breeding selection, PA99N82–4 [23, 25]. The resistance from SB22 is conferred by a dominant allele at single resistance locus R_*MC1(blb)*,_ which is mapped to chromosome 11 [25, 26]. Previously, root penetration assay suggested that nematode resistance in PA99N82–4 is mediated through HR and involves calcium signaling [27]. However, the underlying defense signaling pathway(s) triggered by recognition of nematode effectors by *R*_*MC1(blb)*_ in potato is still largely unknown [26, 28].

In this study, we used PA99N82–4 as a nematode resistant host and commercial variety Russet Burbank, as a susceptible host to provide insights into differential gene expression during the progression of nematode infection in a greenhouse study. Our primary goal is to compare the resistance response to the susceptible interaction based on changes in gene expression during the infection process over the complete life cycle of *M. chitwoodi* and to decipher the triggered plant-pathogen interaction pathways that lead to the resistance response. This study will help potato breeders to better understand the nematode resistance mechanism and design their breeding approaches along with the potential to target CRKN resistance loci with molecular markers in the breeding programs.

## Results

### Time point determination for tissue collection

**Fig. 1:**
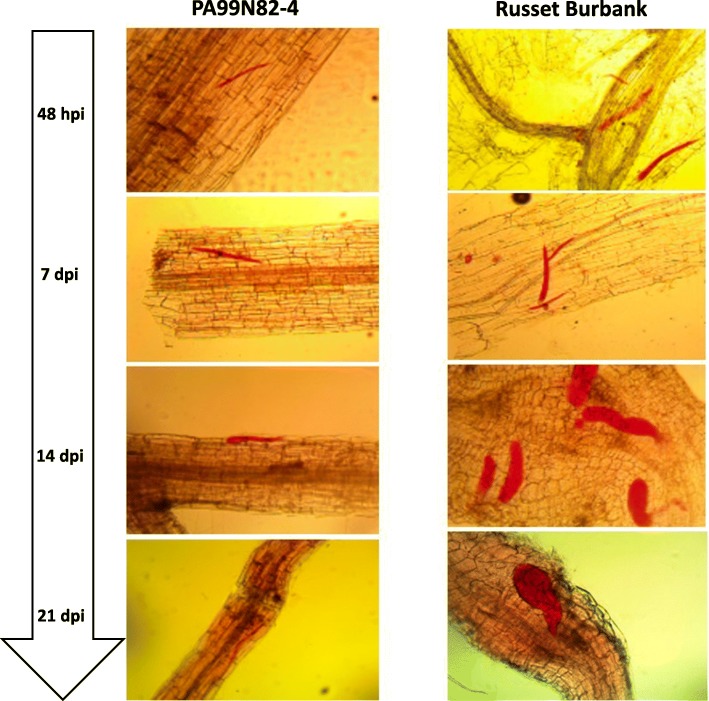
Histological comparison between *Meloidogyne chitwoodi* resistant clone (PA99N82–4) and susceptible clone (Russet Burbank) at 48 h, 7 days, 14 days and 21 days post inoculation. The pictures were taken at 10X resolution

Microscopic evaluations of nematode infected resistant and susceptible potato roots were performed to determine the timeline of nematode infection in compatible and incompatible roots. At 24 h post inoculation, no nematodes were found inside roots of the resistant selection, PA99N82–4 or the susceptible ‘Russet Burbank’ (data not shown). By 48 hpi, nematode juveniles had penetrated both the resistant and susceptible root tissues (Fig. 1). In susceptible roots, some juveniles appeared to have begun feeding shortly after they entered the root tissue (48 hpi); feeding juveniles appeared slightly fatter than non-feeding juveniles. By 14 dpi, nematodes in the susceptible roots had begun to assume their typical sausage-shape, an indication that feeding and molting had progressed to the J3/J4 stage. By 21 dpi, nematodes molted to the adult female stage in ‘Russet Burbank’. The nematode completes its life cycle in 23–25 days in susceptible roots under ideal conditions. In PA99N82–4, the nematodes entered the roots between 24 hpi and 48 dpi, but they did not progress in their life cycle beyond the J2 stage. Although the nematodes were visible inside PA99N82–4 roots at later stages (7 dpi and 21 dpi), no nematode growth or development was observed (Fig. 1). The microscopic analyses confirmed that the nematodes had indeed entered into the resistant host but could not establish feeding sites.

### Transcriptome profiling and differential gene expression

**Fig. 2:**
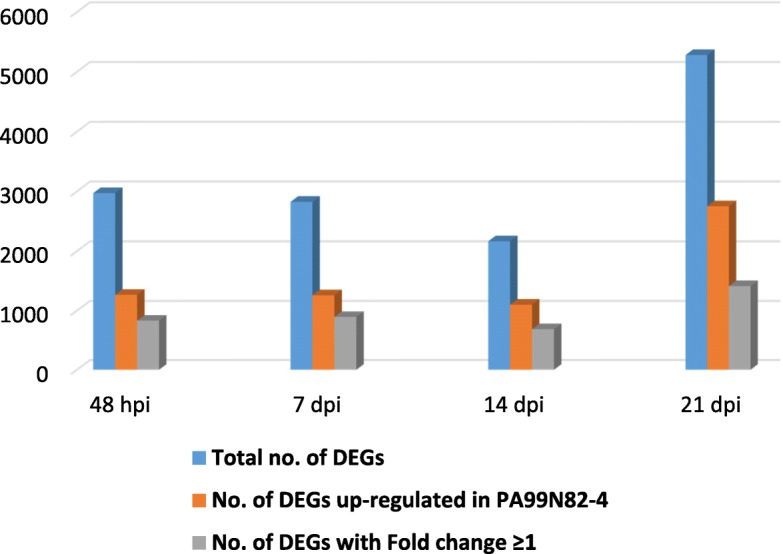
Bar graphs showing summary of all the differentially expressed genes and the genes up-regulated in the resistant clone (PA99N82–4) over four time points (48 h, 7 days, 14 days and 21 days post inoculation)

**Fig. 3:**
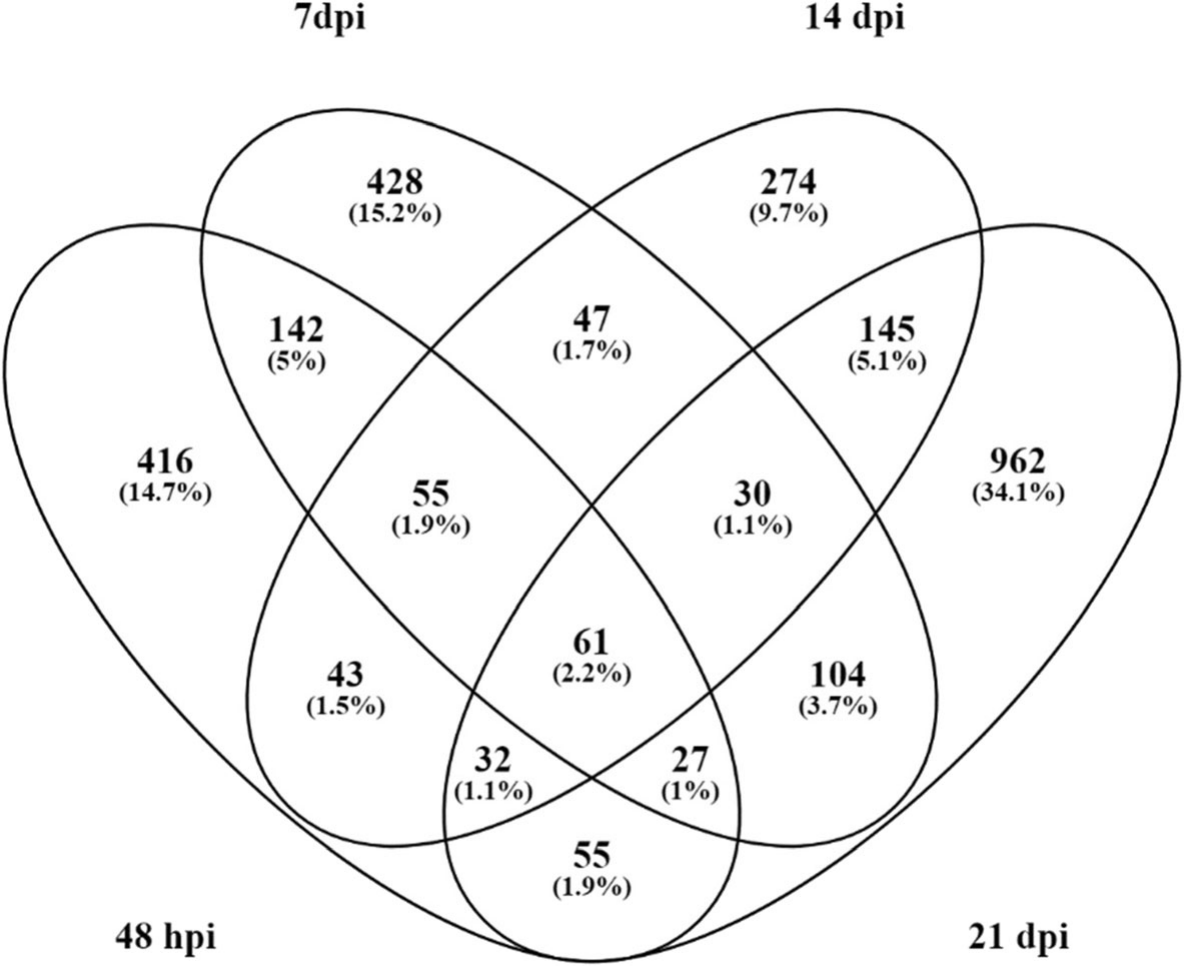
Venn diagram showing the genes common over the four time points and genes specific to four of the time points (up-regulated in PA99N82–4 with FC ≥ 1)

**Fig. 4:**
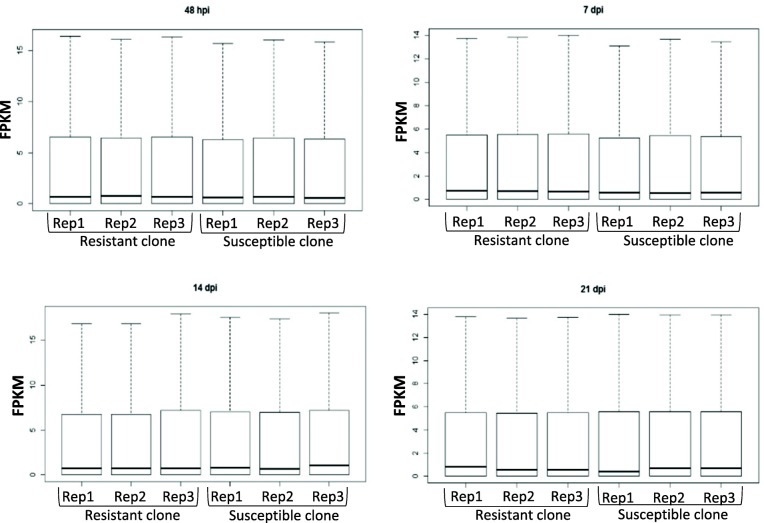
Box plots showing RNAseq data comparison (based on FPKM of differential expressed genes) between three biological replicates each of the resistant clone (PA99N82–4) and the susceptible clone (Russet Burbank) across four time points (48 h, 7 days, 14 days and 21 days post inoculation)

RNAseq of three replicates each of PA99N82–4 and ‘Russet Burbank’ at four different time points resulted in an average of 33 million reads per replicate (Table 1). PA99N82–4 and ‘Russet Burbank’ transcripts were mapped separately to the *S. tuberosum* (Phureja-DM pseudomolecules) reference genome using Hisat2. On average, 78.77% of reads of both the clones mapped to the reference potato genome. Differential gene expression was recorded as the combined FPKM value of the three biological replicates between PA99N82–4 and ‘Russet Burbank’ at each time point using Cuffdiff. The highest number of differentially expressed genes with fold change ≥1 (referred to as significant DEGs hereafter in the text) were recorded at 21 dpi (5282), of which 52.1% (2753) were up-regulated in the resistant clone and the least number of DEGs were recorded at 14 dpi (2166), of which 50.9% (1102) were up-regulated in the resistant clone. An equivalent number of DEGs i.e. 2973 and 2896 were recorded at 48 hpi and 7 dpi, of which 42.6% (1268) and 41.9% (1261) DEGs were up-regulated in the resistant clone, respectively (Table 2 and Fig. 2). Of all the DEGs, only 61 genes (2.2%) were common among all four-time points (Fig. 3). In addition, 24, 25, 23 and 33 genes were expressed only in the resistant clone at 48 hpi, 7 dpi, 14 dpi and 21 dpi respectively. The comparison of three replicates of each library sequenced at each time point suggests that the data generated is of high quality and comparable among all six replicates across each time point (Fig. 4).

**Table 1:** Total number of sequenced reads and the mapping percentage of 24 libraries sequenced using Illumina HiSeq 3000

Library name	Total no. of sequenced reads	No. of mapped reads	Percent mapped reads
PA99N82–4_48 hpi_Rep1	36,006,390	28,658,249	79.70%
PA99N82–4_48 hpi_Rep2	32,535,083	23,590,096	72.68%
PA99N82–4_48 hpi_Rep3	32,015,587	24,903,593	77.87%
PA99N82–4_7 dpi_Rep1	29,687,468	20,689,188	69.78%
PA99N82–4_7 dpi_Rep2	32,428,022	18,773,826	57.79%
PA99N82–4_7 dpi_Rep3	38,735,092	24,514,654	63.30%
PA99N82–4_14 dpi_Rep1	35,725,073	26,963,309	75.50%
PA99N82–4_14 dpi_Rep2	37,050,174	27,659,207	74.74%
PA99N82–4_14 dpi_Rep3	33,921,088	28,152,554	83.10%
PA99N82–4_21 dpi_Rep1	34,488,511	29,721,424	86.29%
PA99N82–4_21 dpi_Rep2	35,811,762	29,629,073	82.84%
PA99N82–4_21 dpi_Rep3	34,541,968	28,757,700	83.36%
Russet Burbank_48 hpi_Rep1	37,167,700	30,221,101	81.41%
Russet Burbank_48 hpi_Rep2	33,674,984	26,881,866	79.92%
Russet Burbank_48 hpi_Rep3	37,027,642	30,005,844	81.14%
Russet Burbank_7 dpi_Rep1	32,641,052	23,764,692	72.84%
Russet Burbank_7 dpi_Rep2	28,964,657	23,553,284	81.41%
Russet Burbank_7 dpi_Rep3	28,367,927	21,524,660	75.98%
Russet Burbank_14 dpi_Rep1	38,250,425	32,461,405	84.99%
Russet Burbank_14 dpi_Rep2	36,979,817	32,044,476	86.76%
Russet Burbank_14 dpi_Rep3	33,964,944	27,090,704	79.85%
Russet Burbank_21 dpi_Rep1	27,859,614	24,711,828	88.81%
Russet Burbank_21 dpi_Rep2	33,289,516	28,329,367	85.20%
Russet Burbank_21 dpi_Rep3	33,375,813	28,370,660	85.10%

**Table 2:** Distribution summary of all the differentially expressed genes (DEGs) across nematode resistant clone PA99N82–4 versus nematode susceptible clone Russet Burbank. Three biological replicates of the clones were used at each time point for RNAseq

Libraries compared (3 Replicates each)	Total no. of DEGs	No. of DEGs up-regulated in PA99N82–4	No. of DEGs up-regulated in PA99N82–4 with Fold change ≥1	No. of DEGs expressed only in PA99N82–4
PA99N82–4_48 hpi versus Russet Burbank_48 hpi	2973	1268	831	24
PA99N82–4_7 dpi versus Russet Burbank_7 dpi	2826	1261	894	25
PA99N82–4_14 dpi versus Russet Burbank_14 dpi	2166	1102	687	23
PA99N82–4_21 dpi versus Russet Burbank_21 dpi	5282	2753	1416	33

### Gene ontology enrichment and pathway search

**Fig. 5:**
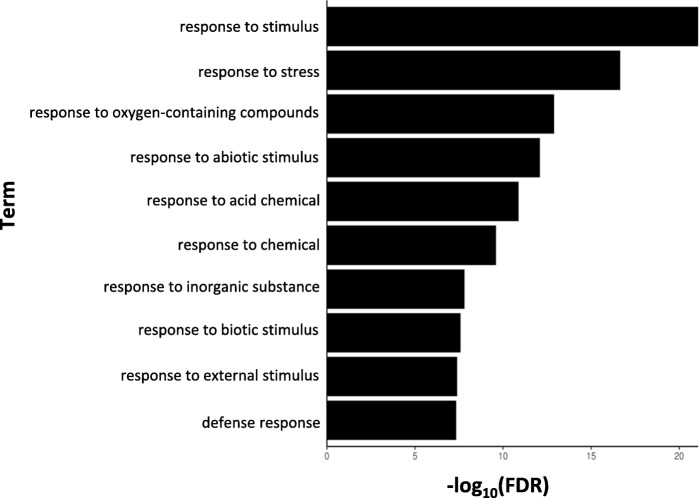
Gene Ontology term enrichments for biological processes of the DEGs up-regulated in nematode inoculated PA99N82–4 at four time points (48 h, 7 days, 14 days and 21 days post inoculation) (PlantRegMap)

**Fig. 6:**
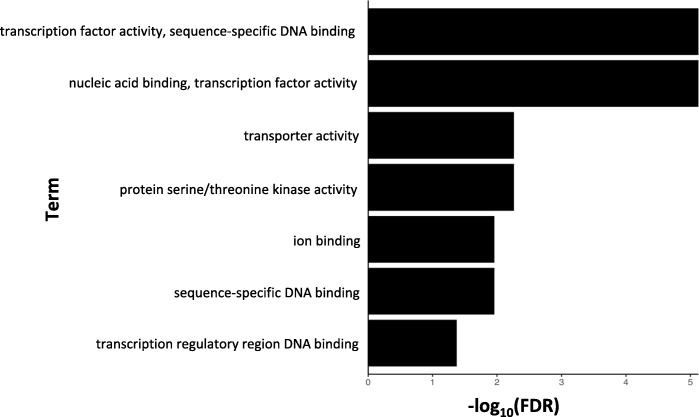
Gene Ontology term enrichments for molecular processes of the DEGs up-regulated in nematode inoculated PA99N82–4 at four time points (48 h, 7 days, 14 days and 21 days post inoculation) (PlantRegMap)

**Fig. 7:**
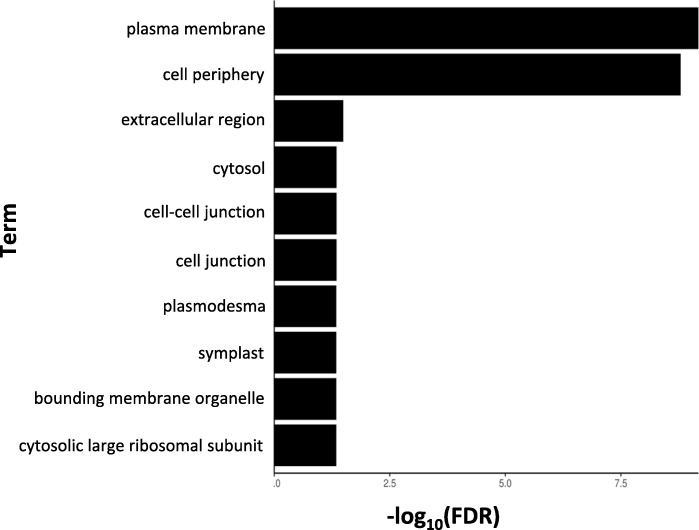
Gene Ontology term enrichments for cellular processes of the DEGs up-regulated in nematode inoculated PA99N82–4 at four time points (48 h, 7 days, 14 days and 21 days post inoculation) (PlantRegMap)

DEGs up-regulated in the resistant clone, PA99N82–4 at four time points were enriched for 265 GO terms (biological, molecular and cellular processes with threshold *p*-value ≤0.01) (Additional file 1). Among the enriched categories were the genes differentially expressed in response to external stimulus, defense response, transcriptional activity, DNA binding and transporter activity (Figs. 5, 6 and 7). KEGG pathway mapping using the genes classified for *S. tuberosum* revealed that in addition to the regular metabolic and developmental pathways, significant DEGs also mapped to the defense related pathways, like plant-pathogen interaction pathways, plant hormone signaling, MAPK signaling, glutathione and flavonoid metabolism, endocytosis and phagosome activity, cell-wall reinforcement and polyamine biosynthesis.

### Significant DEGs and triggered host defense pathways

#### Plant-pathogen interaction pathways

**Fig. 8:**
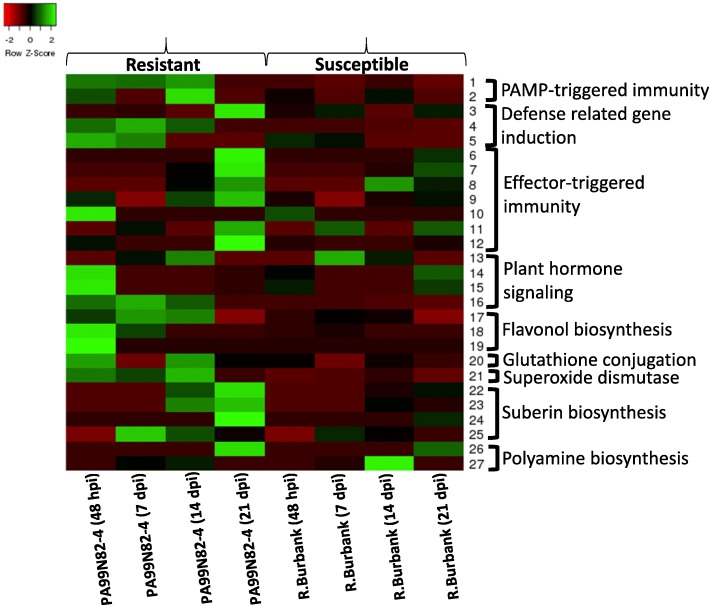
Heatmap showing the expression pattern of all the genes that mapped to various host defense response pathways at 48 h, 7 days, 14 days and 21 days post inoculation

Twenty-seven of the significant DEGs were mapped to plant-pathogen interaction pathways. Similar to previous findings, calcium plays a role in plant defense against the CRKN, with calcium-dependent protein kinase (*CDPK*) and calmodulin-regulated receptor kinase (*CaM)* up-regulated in the resistant clone. Differential gene expression data also showed that *CDPK* expression is up-regulated in the resistant clone as soon as the nematode enters into the root tissue (48 dpi) and stays up-regulated until 14 dpi and the expression level starts to drop at 21 dpi. *CaM* shows an increase in expression in the resistant clone at 48 hpi and 14 dpi. The LRR receptor-like serine/threonine-protein kinase (*FLS2*) is an important defense-related gene whose induction leads to defense responses, including phytoalexin accumulation. *FLS2* was up-regulated in the resistant clone at 48 hpi and the expression further increased at 21 dpi. This gene indirectly leads to ROS accumulation and induction of pathogenesis-related protein 1 (PR-1). Basic PR-1 protein is highly up-regulated in the resistant clone starting at 48 hpi; the expression is highest at 7 dpi and drops at 21 dpi. In addition, pathogenesis-related genes transcriptional activator (*pti6*) was up-regulated in the resistant clone at 48 hpi and 7 dpi (Fig. 8 and Additional file 2: Figure S1).

In the ETI response pathway, known R-genes and genes involved in R-gene mediated resistance were up-regulated in the resistant clone upon nematode infection. These genes include, NBS-LRR disease resistance protein (*RPM1*-like) (up-regulated at 21 dpi), NBS-LRR resistance protein (*RPS2*) (slightly down-regulated at 48 hpi and up-regulated at 21 dpi), protein *SGT1* homolog A-like (up-regulated at 14 dpi and 21 dpi), receptor serine-threonine protein kinase (*PIK1*) (down-regulated at 14 dpi and up-regulated at 21 dpi), enhanced disease susceptibility 1 protein (*EDS1*) (up-regulated at 48 hpi, 14 dpi and 21 dpi) and *HSP90* (up-regulated at 48 hpi). In addition, transcription factor *WRKY* was up-regulated at 48 hpi and 21 dpi (Fig. 8 and Additional file 2: Figure S1).

#### Plant hormonal signal transduction

Jasmonic acid (JA) and salicylic acid (SA) are two plant hormones known to play significant role in plant defense responses. Genes related to JA and SA signaling pathways were up-regulated during infection of the resistant clone. Two genes involved in JA-mediated responses, jasmonate ZIM-domain protein 1 (*JAZ*) was up-regulated at 14 dpi and *MYC2*, was up-regulated at 48 hpi. Similarly, two genes that are activated in response to SA accumulation, *BOP/NPR1/NIM1*-like regulatory protein and basic PR-1 protein were both up-regulated at 48 hpi and all time points thereafter. Basic PR-1 protein is considered as the marker for SA accumulation (Fig. 8 and Additional file 3: Figure S2).

#### Antioxidant activity

Three major genes involved in flavonoid biosynthesis were up-regulated in the resistant clone: Phenylalanine ammonia lyase (*PAL*) over expressed at 48 hpi, 7 dpi and 14 dpi; chalcone synthase (*CHS*) was highly up-regulated at 48 hpi and 7 dpi and flavonol synthase was up-regulated at 48 hpi. In addition, gene for peroxidation of glutathione (a known plant antioxidant), glutathione S-transferase was also up-regulated at 48 hpi, 14 dpi and 21 dpi. The gene that acts as a first line of defense against the over accumulation of ROS, superoxide dismutase (*SOD*) was highly up-regulated at 48 hpi, 7 dpi, 14 dpi and 21 dpi in the resistant clone, indicating the ROS activity as host response in the root tissue (Fig. 8).

#### Cell wall re-enforcement mechanism

Genes involved in cell-wall re-enforcement by deposition of suberin were up-regulated in the resistant clone. Three genes, ER glycerol-phosphate acyltransferase, 3-ketoacyl-CoA synthase, Cytochrome P450 and S-adenosylmethionine-dependent methyl transferase were significantly up-regulated in the resistant clone during the later stages of the progression of infection (14 dpi and 21 dpi) (Fig. 8).

#### Polyamine biosynthesis

Two spermidine biosynthesis pathway genes, arginine decarboxylase and putrescine N-methyltransferase/spermidine synthase that convert arginine into spermidine were up-regulated in the resistant clone at 21 dpi only (Fig. 8).

#### qPCR validation of top differentially expressed transcripts

**Fig. 9:**
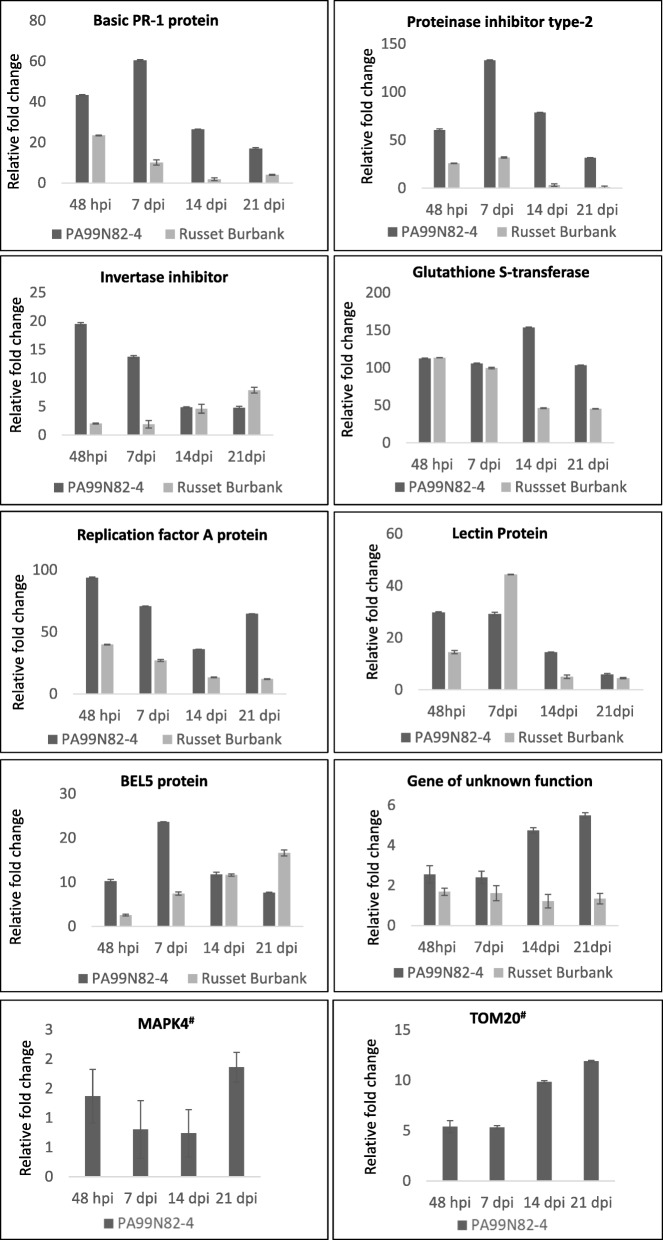
Quantitative reverse transcription polymerase chain reaction (qRT-PCR) validation of significant DEGs (Differentially Expressed Genes with fold change ≥1). X-axis shows the four tissue collection time points and Y-axis shows the relative fold change between PA99N82–4 (resistant clone) and Russet Burbank (susceptible clone) calculated using ^δδ^ct method with the qRT-PCR ct-values. Two technical replicates each of three biological replicates were used for the qRT-PCR. Error bars represent the standard deviation of ct between the biological replicates. ^#^Undetermined in Russet Burbank

**Fig. 10:**
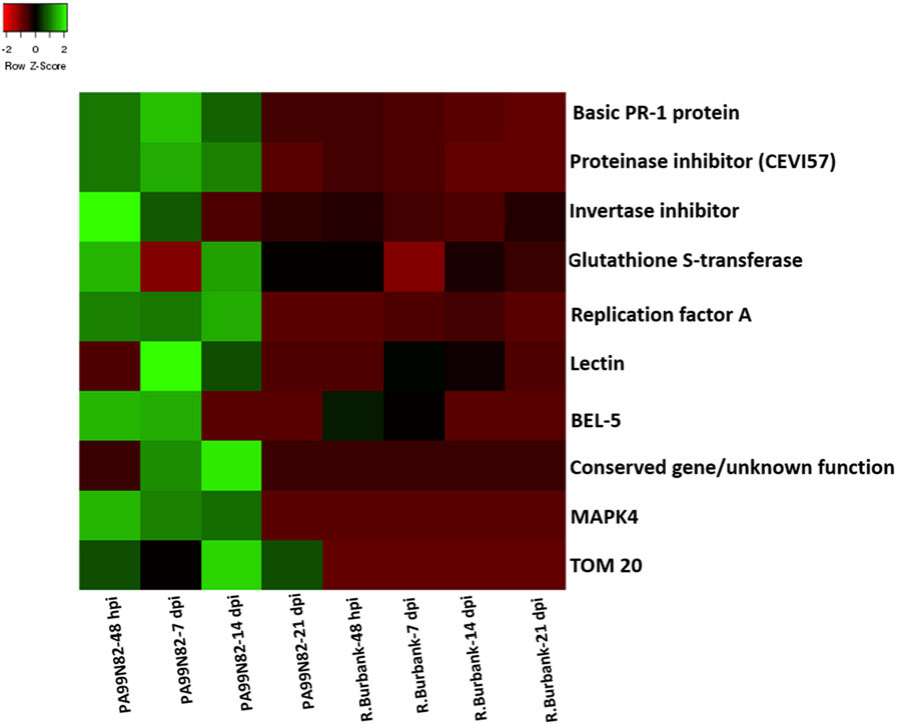
Heat map showing the expression pattern (FPKM) of all genes across all the time points in the resistant clone (PA99N82–4) and the susceptible clone (Russet Burbank) validated using qPCR

qPCR analysis of the top ten significant DEGs showed that the RNAseq data (at four timepoints) for the tested genes corroborate with the qPCR amplification pattern. qPCR fold change difference of ten of the genes differentially expressed (up-regulated in the resistant clone) between the resistant and susceptible clone is presented in Fig. 9 and the RNAseq expression is presented in Fig. 10. These genes include Basic PR-1, glutathione transferase, mitochondrial receptor *TOM20*, mitogen activated protein kinase and *BEL5*. Two of the genes, *TOM20* and *MAPK* showed no expression in the susceptible clone ‘Russet Burbank’ in RNAseq data; these were undetermined in ‘Russet Burbank’ in qPCR as well.

## Discussion

Pathogens including nematodes are known to trigger a host immune response by injecting effector molecules into the host tissue [10]. These effector molecules are small proteins that alter host cell-structure and function [29] and are known to trigger or suppress the host immune response. A resistant host conditions its immune response by reprograming its transcriptional machinery by recognizing pathogen effectors. Next-generation sequencing technologies have enabled accurate comparisons of gene expression between resistant and susceptible genotypes during the progression of pathogen infection. In this study, in-depth analysis of differential gene expression between nematode resistant and susceptible potato clone was performed after *M. chitwoodi* inoculation. The resistant clone, PA99N82–4, is a tetraploid advanced breeding selection known to harbor nematode resistance introgressed from *S. bulbocastanum* (wild, diploid potato species); the susceptible clone is a tetraploid commercial variety Russet Burbank. ‘Russet Burbank’ is highly susceptible to *M. chitwoodi*, which makes it easier to quantify the resistance response histologically. Thus, using ‘Russet Burbank’ as the susceptible control provided us with the opportunity to develop timeline associated with the nematode life cycle.

Although the gene(s) conditioning root resistance to *M. chitwoodi* in PA99N82–4 has been genetically characterized as a single dominant gene [*R*_*MC1(bulb)*_] and mapped to potato chromosome 11, there is limited understanding of the underlying resistance mechanism [25, 26]. The only study using the PA99N82–4 inoculated with *M. chitwoodi* was done by Davies et al., in 2015. They functionally characterized the R_*MC1(blb)*_ mediated resistance response against *M. chitwoodi* using histological characterization of giant cells, quantification of ROS activity and use of chemical ROS inhibitors. In an effort to understand the overall resistance pathway(s) triggered during the nematode infection, we studied gene expression in the roots of resistant PA99N82–4 versus susceptible ‘Russet Burbank’ potato clones at four different time points during the progression of nematode infection. To provide favorable conditions, nematode inoculations were carried out in a 2:1 sand:soil mix in a standard greenhouse set up. Four replicates each of resistant and susceptible clones were inoculated directly with second stage *M. chitwoodi* juveniles (J2 stage) to avoid or surpass the time required for egg hatching. Microscopic observations revealed that juveniles required approximately 48 h after inoculation to enter the root tissue under typical greenhouse conditions. Root tissue was collected at five-time points: 24 hpi, 48 hpi, 7 dpi, 14 dpi and 21 dpi. No juveniles were detected within the root tissue of either the resistant or the susceptible clones at 24 hpi, and hence, we excluded this time point from further analysis. Interestingly, Davies et al. (2015) reported J2’s in the potato root tissue at 24 hpi. They inoculated the nematodes directly onto roots grown in propagation media, making it easier for juveniles to rapidly locate host roots. In our study, juveniles had to move through the sand:soil mix to find the host roots; for that reason we believe it required more than 24 h for juveniles to infect root tissue.

On average, 3000 genes were differentially expressed between the resistant and the susceptible clone at each time point, out of which ~ 50% (fold change ≥1) were up-regulated in the resistant clone. Differentially expressed genes (up-regulated in the resistant clone) were triggered in response to external stimuli such as chemicals, biotic stressors, oxygen containing compounds, and inorganic substances. These genes are known to possess transcriptional activity, DNA and ion binding activity, and transporter activity. In addition, these genes are also known to function in extracellular regions such as the cell periphery, cell-cell junction, cytosol, symplast and plasmodesmata. Therefore, differential expression is due to the presence of external stimuli, which could include nematode secretions; the host responds by activating its immune response. The majority of the differentially expressed genes mapped to primary metabolic pathways, host-pathogen interaction pathways, plant hormone signaling, mitogen activated protein kinases (*MAPK*) signaling and secondary metabolite metabolism. Host-pathogen interaction pathways were similar to those triggered in response to external stimuli, such as bacterial-flg22, fungal-Avr9 and other bacterial secretions.

Plant defense response consists of two major pathways: PAMP-triggered immunity (PTI) and effector-triggered immunity (ETI). These pathways are interconnected and activate local as well as systemic acquired resistance (SAR) responses in the resistant host, which is modulated by two major plant hormones, SA and JA [30]. ETI is known to enhance the pathways initiated as PTI response including mobilization of Ca^2+^ dependent and mitogen-activated protein kinases, production of ROS and accumulation of SA [31, 32]. Our transcriptome data indicate that genes with roles in PTI and ETI are differentially up-regulated in the resistant potato roots during nematode infection. For example, the flagellin22 activated serine/threonine protein kinase (*FLS2*), is a host receptor involved in PTI; this gene is up-regulated in the resistant clone during infection. *FLS2* perceives bacterial pathogen-associated molecular patterns (PAMPs), and the gene is typically up-regulated in expression during bacterial attack [33]. It is possible that potato *FLS2* is capable of detecting unknown nematode PAMPs or bacteria stuck to the nematode cuticle to elicit PTI and contribute to overall plant defenses against nematodes. ETI against root-knot nematodes is well documented in resistant tomatoes carrying the single dominant resistant gene *Mi 1.2*. When nematodes try to establish a feeding site in the resistant tomato roots, they elicit an HR around the head of the nematode [34]. This resistance triggered by *Mi1.2* in tomato roots shows similarities to the resistance seen in the PA99N82–4 roots in the present study. There is evidence that the *Mi*-mediated resistance is dependent on SA [35, 36]. Interestingly, our transcriptome data also suggest that SA may play an important role in plant resistance against nematodes with an up-regulation of SA-regulated marker genes, *BOP/NPR1/NIM1*-like and basic PR-1 during nematode attack [37–40].

Previous work involving root penetration assay in PA99N82–4 reported HR around the head of nematode juveniles, suggesting that the nematode triggers a strong defense response while attempting to establish feeding sites, at around 7dpi [27]. The study also implicated the role of calcium in the resistance response. If nematode juveniles fail to establish a feeding site, they eventually die because of the dearth of nutrients required for growth and development. Our histological data suggest that juveniles entered root tissues of both resistant and susceptible clones. However, in the resistant clone PA99N82–4, nematode juveniles failed to develop further. Our gene expression analysis also shows induction of calcium related genes [calcium-dependent protein kinase (*CDPK*) and calmodulin-regulated receptor kinase (*CaM*)] in addition to genes that are related to ROS production and HR in the resistant roots. For example, genes involved primarily in regulation of ROS, such as superoxide dismutase (*SOD*) and glutathione transferase were up-regulated in PA99N82–4 roots after nematode infection. These are a major part of the scavenging system that clears the free radicals after HR and act as antioxidants that protect the host tissue from further damage. In addition, phenylalanine ammonia lyase (*PAL*) and chalcone synthase B (*CHS*), the major genes involved in the phenylpropanoid and flavonoid pathways, were up-regulated during the resistance response. These genes are known to be induced by wounding, salinity stress, and pathogen attack [41, 42] and constitute the secondary antioxidant (ROS scavenger) system, which is activated after the depletion of primary antioxidant enzymes [43]. *CHS* up-regulation would indicate oxidative stress in resistant roots. Altogether, our data support the hypothesis that CRKN infects resistant PA99N82–4 roots but triggers strong defense responses as it attempts to establish a feeding site. In addition, *PAL* indicates a SA-accumulation during the resistance response.

Plant resistance proteins (R-protein) often contain putative nucleotide-binding sites (NBS) and leucine-rich repeats (LRR) domains. When we searched our transcriptome data for R-genes and their signaling partners, we found *RPM1*, *RSP2*, *SGT1*, *PIK1*, *EDS1*, *HSP90* up-regulated in PA99N82–4. Although R-genes *RPM1*, *PIK1*, and *RSP2* are not known to be involved in nematode resistance, the upregulation of these genes suggests that transcriptional control of R-genes in general may be released, allowing for their enhanced expression [44]. The enhanced expression of R-gene co-chaperones *SGT1* and *HSP90* also points to a modulation of R-protein levels in resistant roots. *EDS1* is known to be involved in signal amplification and protection of SA-dependent defense response pathways [45]. In addition, transcription factor *WRKY* was also up-regulated in PA99N82–4. Members of the *WRKY* gene family exhibit functional redundancy and the contribution of individual members in a resistance response is indistinct. *WRKY* genes have been indicated to play a significant role in *Mi-1* mediated gene-for-gene resistance response to bacterial pathogen in *Arabidopsis* [46] and *Mi-1* mediated resistance to aphids and nematodes in tomato [47]. Recently, WRKY genes have been shown to enhance soybean cyst nematode resistance in transgenic soybean lines overexpressing three of the WRKY genes [48]. It is clear from the gene expression analysis that PA99N82–4 contains a single dominant resistance gene that elicits a strong HR however; this resistance gene is yet to be identified.

Up-regulation of the genes involved in polyamine biosynthesis during the resistance response in PA99N82–4 is interesting as conjugates of polyamine such as spermine and spermidine have been reported to accumulate during the activity of the plant resistance mechanism to various pathogens [49, 50]. Researchers observed the accumulation of conjugated forms of spermine and spermidine in barley at 1–4 days after inoculation with powdery mildew and suggested that these metabolites are involved in the development of the HR [51]. More recently, Goyal et al. (2016) proposed that polyamines, spermine and spermidine, in combination with cold stress upregulate *PRb1* in tomato, and thereby contribute to cold stress induced disease resistance [52]. Higher PA levels have been detected in plant tissues exposed to biotic stresses [53, 54]. PAs act as scavengers of ROS to prevent damage to host tissue during stress tolerance [55, 56], however their role in nematode resistance is unknown. Based on our transcriptome data, polyamine biosynthesis is induced in resistant roots, and correlates with the nematode resistance response; however, the mode of action of these polyamines still must be investigated.

Cell-wall reinforcement by the deposition of cell-wall constituents has been observed to be PAMP induced, and occurring as a late response to various pathogens [57]. In PA99N82–4 infected roots, up-regulation of genes involved in suberin biosynthesis was observed. Interestingly, suberin is a component of the Casperian strip of the root epidermis and suberized cells are known to act as a transport barrier limiting the movement of water and nutrients, and protecting plant cells from pathogen invasions [58].

### Proposed resistance response model

**Fig. 11:**
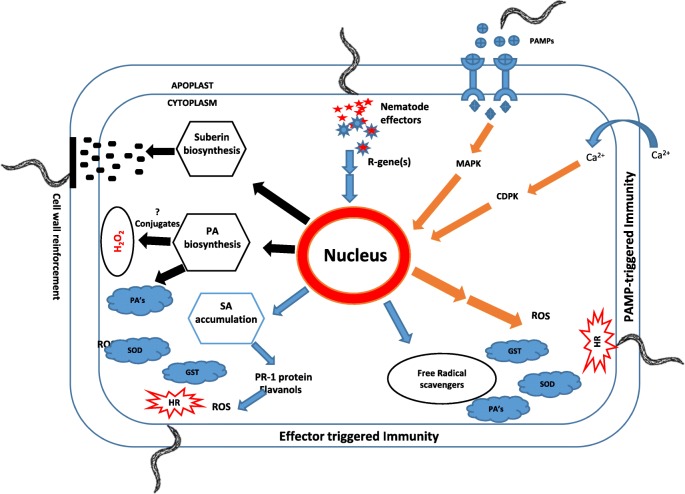
Proposed model describing the mechanism of resistance response occurring in PA99N82–4 that contains *Meloidogyne chitwoodi* resistance introgressed from *Solanum bulbocastanum*. PAMP-triggered immunity is indicated in orange, effector-triggered immunity is indicated in blue and other secondary processes are indicated in black

Our proposed model of plant-nematode resistance interaction suggests that J2’s enter the roots of both resistant and susceptible potato plants and the nematodes migrate to the vasculature where they attempt to establish feeding sites. With nematodes in and around resistant host root tissue, PTI is triggered as an early response. Subsequently, when nematodes migrate more deeply into the root vasculature and secrete a suite of molecules (effectors) to initiate feeding site formation, one or more of these effectors are recognized by the R-gene(s) present only in the resistant host. This interaction between the nematode effector and host R-gene activates gene expression leading to ETI. ETI triggers the accumulation of SA, which subsequently results in ROS accumulation and HR. We believe that ETI based HR inhibits feeding site formation and thus nematodes fail to develop further. Eventually, juveniles die or migrate out of the root system. The resistance response also activates the ROS scavenging system in the host. It seems that primary and secondary scavenging systems alike are activated to lessen or prevent the impact of ROS activity on the host cells. The role of polyamines in resistance response mechanisms warrants further research; it may act as both HR mediator and the ROS scavenger. We also hypothesize that suberin plays a crucial role in cell wall re-enforcement of the resistant host root tissue to prevent it from further nematode attacks (Fig. 11). Ultimately, additional work will be required to characterize the specific roles of up-regulated candidate genes in the *M. chitwoodi* resistance mechanism in PA99N82–4. Once these genes are validated, the data can be used to develop molecular markers linked to the resistance trait to facilitate marker-assisted-selection for development of CRKN resistant potato varieties for the PNW potato production region of the United States.

## Conclusion

Columbia root-knot nematode (*Meloidogyne chitwoodi*) is a potato pest of economic significance in the Pacific Northwest of the United States of America. It negatively impacts potato yield and tuber quality. Current control practices are limited to the use of hazardous chemical fumigants and nematicides. Development of nematode resistant potato varieties could be a far more effective approach to reduce damage to the crop, but resistant potato varieties for commercial distribution are unavailable. Resistance to this nematode was identified in wild potato species and later bred into advanced potato selection, but the underlying resistance mechanism is largely unknown. Based on histological and gene expression data, the nematode can enter both resistant and susceptible potato roots, but the resistant plant inhibits feeding site formation, a major event in nematode parasitism. The presence of the nematode inside the resistant root tissue triggers an immunological response that restricts further development of the nematode. This is the first-ever report of gene expression analysis characterizing the resistance response to CRKN in potato. The knowledge generated by this study has implications for potato breeding, thus reducing chemical inputs to the crop, and easing the environmental impacts of potato production.

## Methods

### Plant material and nematode inoculum

Tissue culture plantlets of *M. chitwoodi* (race 1) resistant breeding clone PA99N82–4 and susceptible cultivar Russet Burbank were procured from the Potato Tissue Culture Lab (Nuclear Seed Potato Program), University of Idaho, Moscow, Idaho, USA. Plantlets were grown for four weeks in a 2:1 sand: soil mixture in one-gallon clay pots in tightly regulated greenhouse conditions (18.5 °C and 20 h light). *M. chitwoodi* race 1 eggs were acquired from the United States Department of Agriculture, Agriculture Research Service, Prosser, Washington, USA. Eggs were extracted from 10-week old infected tomato roots using 40% bleach solution, suspended in distilled water and held in petri dishes for ten days at 24 °C under dark condition to promote hatching. At regular intervals, 1 ml of the hatching solution was applied to a hemocytometer and observed under a microscope for juveniles. Subsequently, hatched second stage juveniles (J2) were counted and stored in glass bottles at 4 °C.

### Nematode inoculation and tissue collection

Four replicates each of PA99N82–4 and ‘Russet Burbank’ were included for each of five different time points: 24 hpi, 48 hpi, 7 dpi, 14 dpi and 21 dpi. The replicates were inoculated with 1200 freshly hatched J2’s each by pipetting J2 suspension in equidistant shallow holes made around the root surface. Root tissue of three of the replicates was collected for RNAseq studies and one replicate was subjected to microscopic examination in order to determine the progression of the infection. Whole root tissue was washed thoroughly under running tap water, dried carefully with paper towels, snap-frozen in liquid nitrogen and stored at − 80 °C until RNA isolation.

### Microscopic examination

Microscopic examination of the inoculated root tissue (24 hpi to 21 dpi) was performed to confirm the progression of infection and to select the time points for RNAseq. Roots were washed thoroughly under running tap water and stained with fuchsin-glycerin as described by Bybd [59]. Roots were cut into tiny (~ 1 cm) pieces and stained by boiling in acid fuchsin (3.5 g acid fuchsin, 250 ml acetic acid and 750 ml distilled water) for one minute, and de-stained by boiling in glycerin for one minute. De-stained root tissue was then cooled to room temperature, observed under a light microscope (10X) (Amscope, Irvine, California, USA), and photographed using an Amscope camera (Amscope, Irvine, California, USA) with Toupview software (Amscope, Irvine, California, USA) at 10X.

### RNA extraction and rRNA depletion

Three biological replicates each of nematode inoculated PA99N82–4 and ‘Russet Burbank’ at time points: 48 h, 7 days, 14 days and 21 days post inoculation were used for RNA extraction. Total RNA was extracted from whole root tissue using the Plant RNA Maxi kit (Omega Bio-tek, Georgia, USA) following the manufacturer’s protocol. Approximately 7–12.5 g of each root was ground thoroughly in liquid nitrogen using RNase-free pestle and mortar. Lysate was transferred through homogenization RNA maxi column, followed by RNA precipitation with absolute ethanol. The precipitated mix was then applied to HiBind RNA maxi spin column and membrane bound RNA was washed several times with the RNA wash buffers provided in the kit. RNA was eluted from the column membrane with RNase-free diethyl pyrocarbonate (DEPC) treated water and stored at − 80 °C. RNA integrity was checked by running a bleach agarose gel [60]; and initial concentrations were checked using the NanoDrop (spectrophotometer) (Thermo Fisher Scientific, Massachusetts, USA). RNA integrity and concentrations were later confirmed using Nano chip in the Agilent Bioanalyser 2100 (Agilent Technologies, Santa Clara, California, USA) at the Center for Genome Research and Biocomputing (CGRB), Oregon State University, Corvallis, Oregon. Ribosomal RNA (rRNA) was depleted using RiboMinus™ Plant Kit (Invitrogen, California, USA) with slight modifications: 5 μg of total RNA was hybridized with the rRNA probes provided in the kit. Hybridization was set at 75 °C for 10 min and cooled at 37 °C over a period of 30 min. Probes were removed using the magnetic beads provided in the kit. The hybridization step was repeated to completely deplete any undesired rRNA. Purified mRNA was precipitated, re-suspended and stored at − 80 °C. mRNA concentrations were checked using Qubit RNA HS Assay kit (Invitrogen, California, USA) and running the samples through a highly sensitive Qubit Fluorometer (Invitrogen, California, USA). Samples showing > 5% rRNA contamination and/or lower than 25 ng/μl of final mRNA concentrations were reprocessed.

### Library preparation and sequencing

Library preparation and sequencing was performed at the CGRB, Oregon State University, Corvallis, Oregon using NEBNEXT® ULTRA™ RNA Library Prep Kit (New England Biolabs, Ipswich, Massachusetts, USA). The libraries were sequenced using an Illumina Hiseq3000 instrument (1X150bp) (Illumina, San Diego, California, USA).

### Differential gene expression analysis

**Fig. 12:**
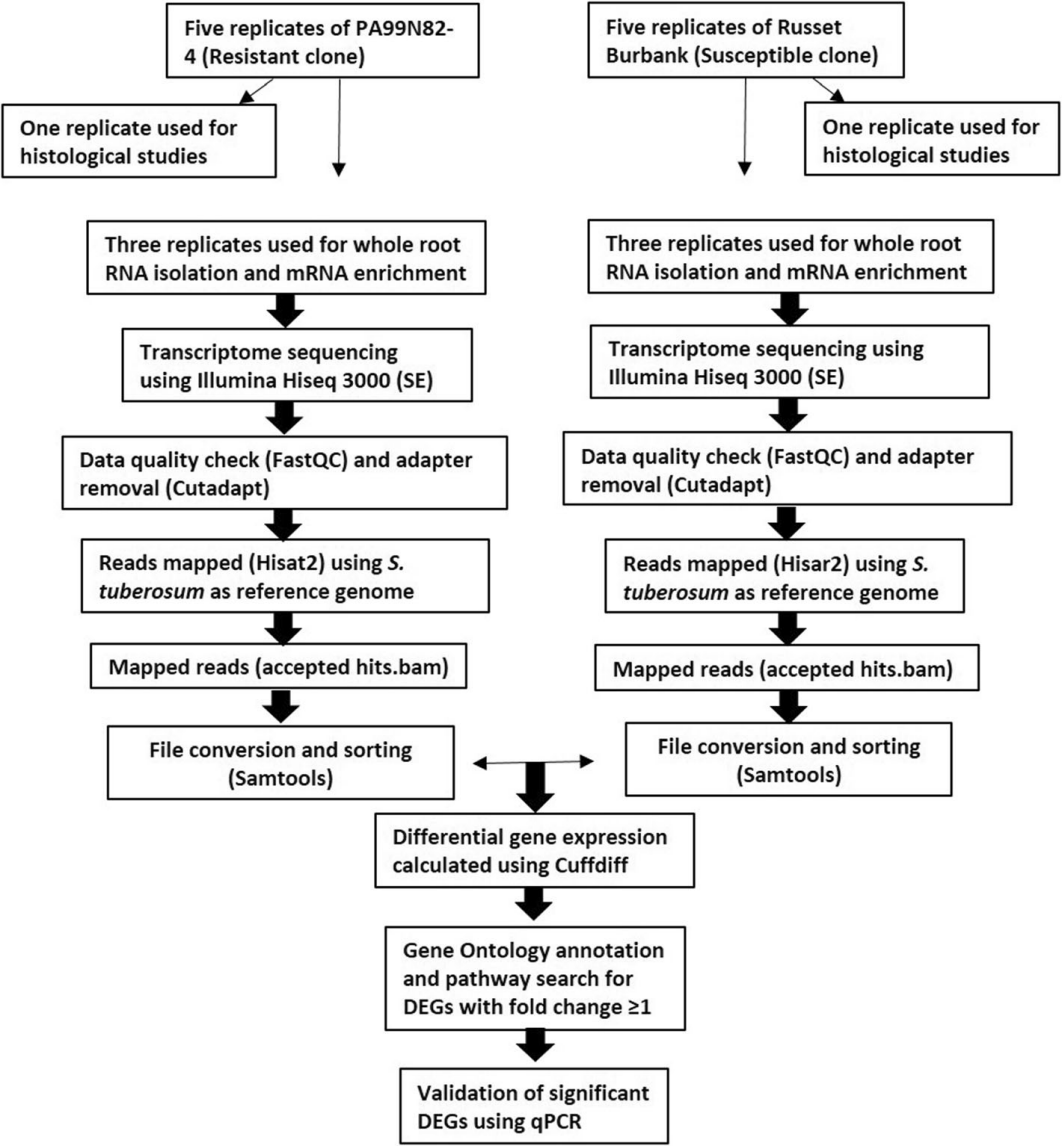
Schematic representation of the methodology (bioinformatics pipeline) used to generate significant differentially expressed gene dataset in the present study

Raw data quality was assessed using FastQC [61] with default parameters (pvalue > 0.01, phred score < 2, error rate < 0.2%, sequence quality > 10, duplicate sequences< 20%). The sequence data was analyzed using a modified version of Tuxedo pipeline [62]. Briefly, adapter sequences from the raw reads were trimmed using Cutadapt and the reads > 25 bp from both the resistant clone PA99N82–4 and the susceptible variety ‘Russet Burbank’ were mapped to *Solanum tuberosum* reference genome (Group Phureja DM1–3 v3.4) using Hisat2. Differential gene expression analyses were performed using Cuffdiff. Fragments per kilo base per million mapped reads (FPKM) was calculated for each transcript in three replicates each of PA99N82–4 and ‘Russet Burbank’ considering the fold change (FC) ≥ 1 as significant. Heatmaps were prepared using Heatmapper [63]. Three replicates each of the resistant and the susceptible clones were compared at each time point based on the FPKM values with graphics produced using ggplot2. The detailed pipeline used for RNAseq data analyses is summarized in Fig. 12.

### Gene ontology and pathway analysis

Gene ontology (GO) categories were assigned to the differentially expressed genes (FC ≥ 1) based on annotations in the PlantTFDB 4.0 [64] using RegMap GO enrichment tool. Using *Solanum tuberosum* as preferred species; GO terms were searched for three major aspects: biological processes, molecular functions and cellular components with threshold *p*-value ≤0.01. In order to perform the pathway search, *S. tuberosum* gene IDs (*PGSCDMG*) of significant genes were converted to Uniprot IDs using gProfiler [65]. Uniprot IDs were converted to KEGG IDs using KEGG ID convert. Subsequently, pathway enrichment analysis was performed with KEGG mapper using *S. tuberosum* as the reference species [66].

### Gene expression validation using qPCR

qPCR validation was performed using the top ten significant genes up-regulated in the resistant clone PA99N82–4 with respect to the susceptible cultivar ‘Russet Burbank’. The shortlisted gene sequences were downloaded from Phytozome (https://phytozome.jgi.doe.gov/pz/portal.html) and primers were designed using Oligo Analyzer 3.1 (Integrated DNA Technologies, Iowa, USA) with the following parameters: Tm: 55–60 °C, Length: 12–30, GC content: 40–58% with no secondary structures. Primers were synthesized from Integrated DNA Technologies (Integrated DNA Technologies, Iowa, USA). Details of the primers is presented in Table 3. A total of 2 μg RNA was processed for DNase treatment to eliminate genomic DNA using a TURBO DNA-free™ kit (Invitrogen, California, USA) according to the instruction manual. Two hundred and fifty nanograms of DNA-free total RNA was used for cDNA synthesis using Tetro Reverse transcriptase kit (Bioline, London, UK) following the instruction manual. The final reaction contained 0.5 μM Oligo [dT]_18_ (Integrated DNA Technologies, Iowa, USA), 1 mM dNTPs (Bioline, London, UK) and PCR grade water. cDNA diluted to 1/5 times provided a template for qPCR amplifications using Quant Studio 3 Real-Time PCR system (Applied Biosystems, Foster city, California, USA). Two technical replicates of each of the three biological replicates along with no RT control (NRT) and no template control (NTC) were used in qPCR reaction for each transcript. The 26S proteasome regulatory subunit (RPN7) gene was used as endogenous control [67]. qPCR data was analyzed with a custom excel spreadsheet. Fold change was calculated using the comparative ^δδ^ct method [68].

**Table 3:** Summary of top ten gene primers used in qPCR validation of the transcriptomic data generated in the present study

Gene code	Gene Name	Forward Primer	Reverse Primer	Tm (°C)
Gene26	Basic PR-1 protein	CTG TAG GAT GCA ACA CTC TGG TGG C	CCA AGA CGT ACT GAG TTA CGC CAG	58
Gene17	Proteinase inhibitor type-2	CAA GGC ATG TAC CCT GGA ATG TGA C	GGC TCT CCA GTA CAA ATT AAA GAT CCA TC	57
Gene 19	Invertase inhibitor	GAT GGT ATG GAT GAT GTT GTT GTT GAA GC	GCA ACT TTT GAT AGT TCA ATT ATT TCC CTA CTC	57
Gene25	Glutathione S-transferase	CTG ATC CTT ATG AGA GAT CAC AAG CC	GCT TCC TCC AGT AAC TTG AGT GG	57
Gene40	Replication factor A protein	GCA CAA ATG TCA TCA GCA GCT TC	GCA TCC TGA GCA TTC AAG CAC	57
Gene89	Lectin protein	GAA GTG GCT GAG CTT GTT AGA ACT TG	GCC TTT TCA AGT CCA TGT GAA TCC TC	58
Gene100	BEL5 protein	GTG GAT CAA AGG TAT AGA CAA TAC CAT CAC C	GAA ATT GTG TGC AAA GCA AGT TGT GTG	58
Gene53	Gene of unknown function	CAC CAC GTA GAT CCC TCT ACC TTA G	CAT GAT CCA CGA TCA GGT GAC G	58
Gene32	Mitogen-activated protein kinase 4	CGG AGA ATC TAC GAG CAG TGG CG	CAC AGA TTC CCT CCA AAT GAG CTC C	57
Gene43	TOM20	CTT GGC GAG GTG GGG ACG	CCC AAA CAC CAA AGC ACA TCA TGC	60

## Supplementary information

**Additional file 1.** Gene Ontology results of all the enriched terms for the genes up-regulated in the resistant clone, PA99N82–4 at 48 hpi, 7 dpi, 14 dpi and 21 dpi.

**Additional file 2: Figure S1.** Schematic representation of plant-pathogen interactions taking place during the nematode resistance response based on the differentially expressed genes (up-regulated in PA99N82–4). All genes colored red are significantly up-regulated in PA99N82–4; green colored genes have been characterized for *Solanum tuberosum* in KEGG pathway. The image was generated using KEGG Mapper search and color pathway tool found at https://www.genome.jp/kegg/tool/map_pathway2.html.

**Additional file 3: Figure S2.** Schematic representation of plant hormone signal transduction taking place during the nematode resistance response based on the differentially expressed genes (up-regulated in PA99N82–4). All genes colored red are significantly up-regulated in PA99N82–4; green colored genes have been characterized for *Solanum tuberosum* in KEGG pathway. The image was generated using KEGG Mapper search and color pathway tool found at https://www.genome.jp/kegg/tool/map_pathway2.html.

## Data Availability

The datasets generated in the present study are included in the manuscript and in the Additional files. Transcriptomic data supporting the conclusions of this article is available in Bioproject#PRJNA580296 (Accession# SAMN13154920 to SAMN13154943) at NCBI (http://www.ncbi.nlm.nih.gov/bioproject/580296).

## Abbreviations

CDPK: Calcium-dependent protein kinases
CGRB: Center for genome research and biocomputing
CRKN: Columbia root-knot nematode
DEG’s: Differentially expressed genes
dpi: Days post inoculation
ETI: Effector-triggered immunity
FC: Fold change
FPKM: Fragments Per Kilobase of transcript per Million mapped reads
GO: Gene ontology
hpi: Hours post inoculation
HR: Hypersensitive response
JA: Jasmonic acid
KEGG: Kyoto encyclopedia of genes and genomes
MAPK: Mitogen-activated protein kinases
mRNA: Messenger RNA
NBS-LRR: Nucleotide-binding site leucine-rich repeats
PA’s: Polyamines
PNW: Pacific Northwest
PTI: PAMP-triggered immunity
R-gene: Resistance gene
RLK’s: Receptor-like kinases
RLP’s: Receptor-like proteins
ROS: Reactive oxygen species
SA: Salicylic acid
