# Correction to: Transcriptome profiling of resistance response to *Meloidogyne chitwoodi* introgressed from wild species *Solanum bulbocastanum* into cultivated potato

**DOI:** 10.1186/s12864-019-6403-9

**Published:** 2020-01-09

**Authors:** Sapinder Bali, Kelly Vining, Cynthia Gleason, Hassan Majtahedi, Charles R. Brown, Vidyasagar Sathuvalli

**Affiliations:** 1 Department of Plant Pathology, Washington State University, Pullman, Washington 99164 USA; 2 Department of Horticulture, Oregon State University, Corvallis, Oregon 97330 USA; 3 Retired from United States Department of Agriculture, Prosser, Washington 99350 USA; 4 Hermiston Agricultural Research and Extension Center, Oregon State University, Hermiston, Oregon 97838 USA

**Correction to: BMC Genomics (2019) 20:907**

**https://doi.org/10.1186/s12864-019-6257-1**

Following the publication of this article [1], the authors noted an error in Fig. 11. Due to a figure processing error two labels were mistakenly retained in the original figure. They are highlighted in red boxes in the erroneous figure below:

**Fig. 11:**
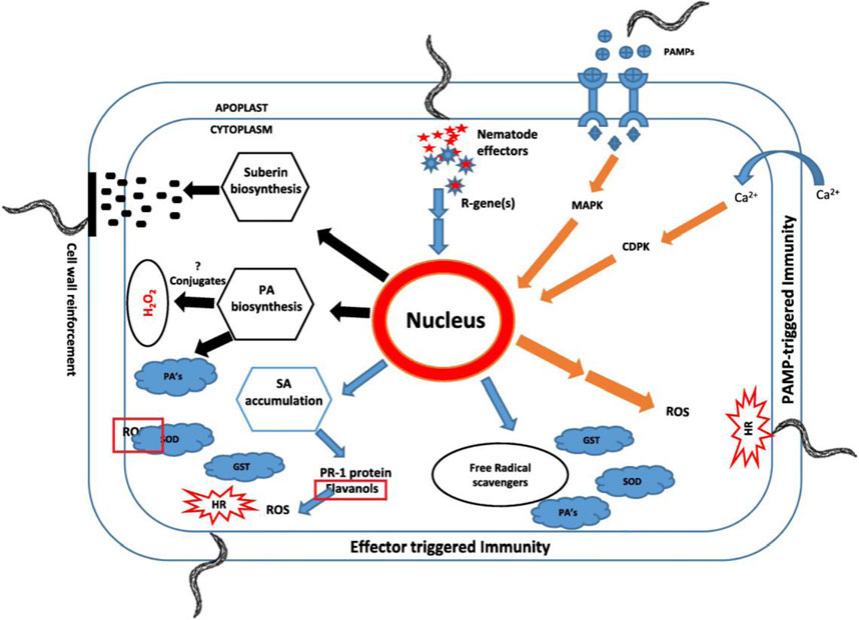
Proposed model describing the mechanism of resistance response occurring in PA99N82–4 that contains Meloidogyne chitwoodi resistance introgressed from *Solanum bulbocastanum*. PAMP-triggered immunity is indicated in orange, effector-triggered immunity is indicated in blue and other secondary processes are indicated in black

The correct figure is:

**Fig. 11:**
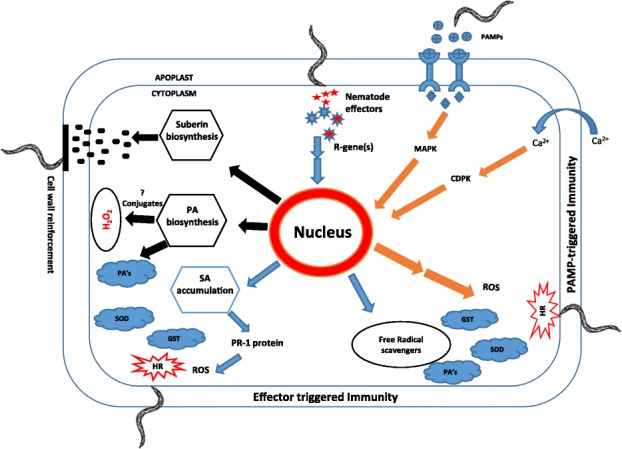
Proposed model describing the mechanism of resistance response occurring in PA99N82–4 that contains Meloidogyne chitwoodi resistance introgressed from *Solanum bulbocastanum*. PAMP-triggered immunity is indicated in orange, effector-triggered immunity is indicated in blue and other secondary processes are indicated in black

The original article has been corrected.
