# Supplementary figures and images for: Transcriptome profiling of resistance response to Meloidogyne chitwoodi introgressed from wild species Solanum bulbocastanum into cultivated potato
Source: BMC Genomics. 2019 Nov 28;20:907. doi: 10.1186/s12864-019-6257-1 (PMC6883582; doi:10.1186/s12864-019-6257-1)

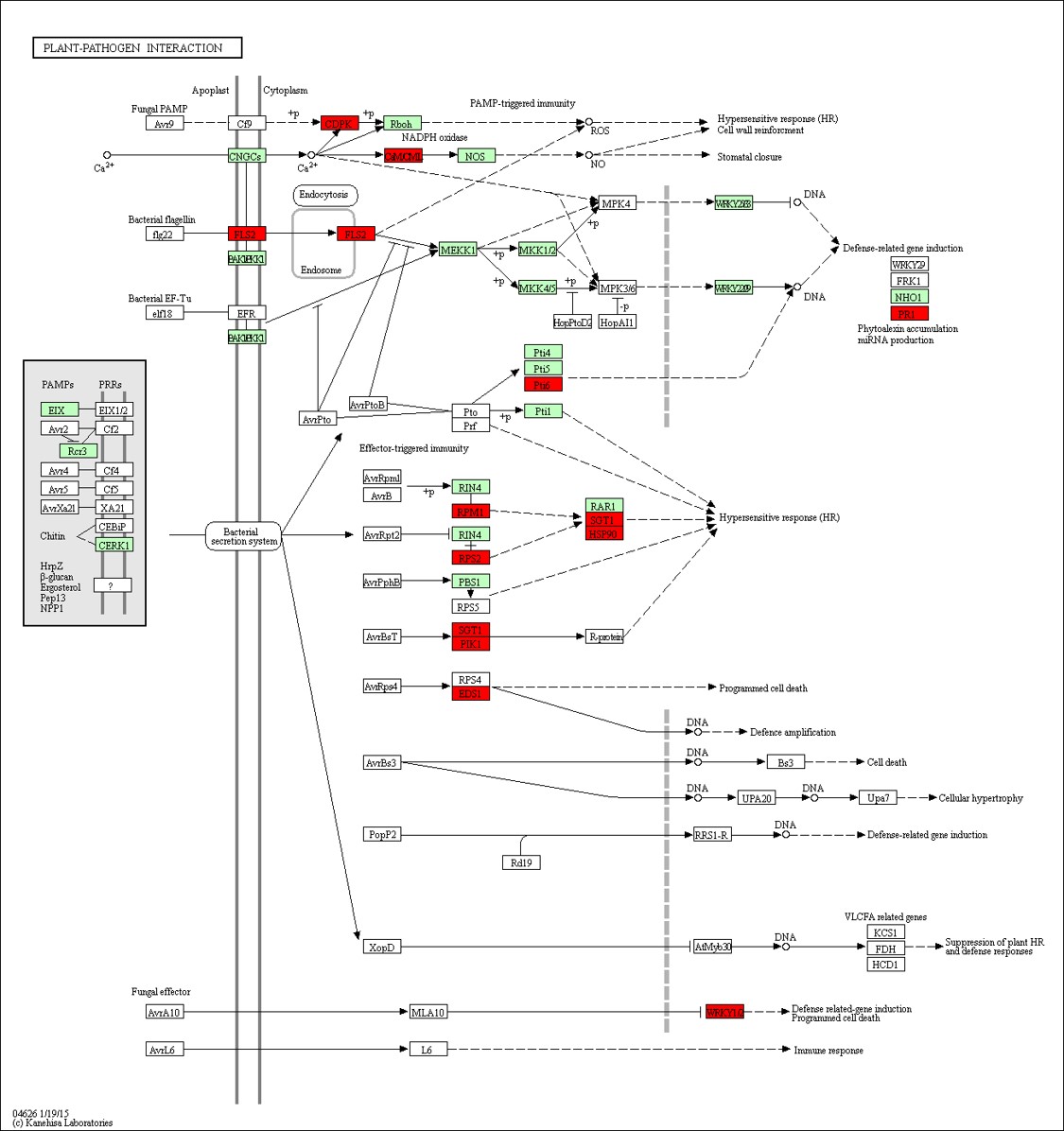

Supplement: Supplementary file 2 — Additional file 2: Figure S1. Schematic representation of plant-pathogen interactions taking place during the nematode resistance response based on the differentially expressed genes (up-regulated in PA99N82–4). All genes colored red are significantly up-regulated in PA99N82–4; green colored genes have been characterized for Solanum tuberosum in KEGG pathway. The image was generated using KEGG Mapper search and color pathway tool found at https://www.genome.jp/kegg/tool/map_pathway2.html. [file 12864_2019_6257_MOESM2_ESM.jpg]

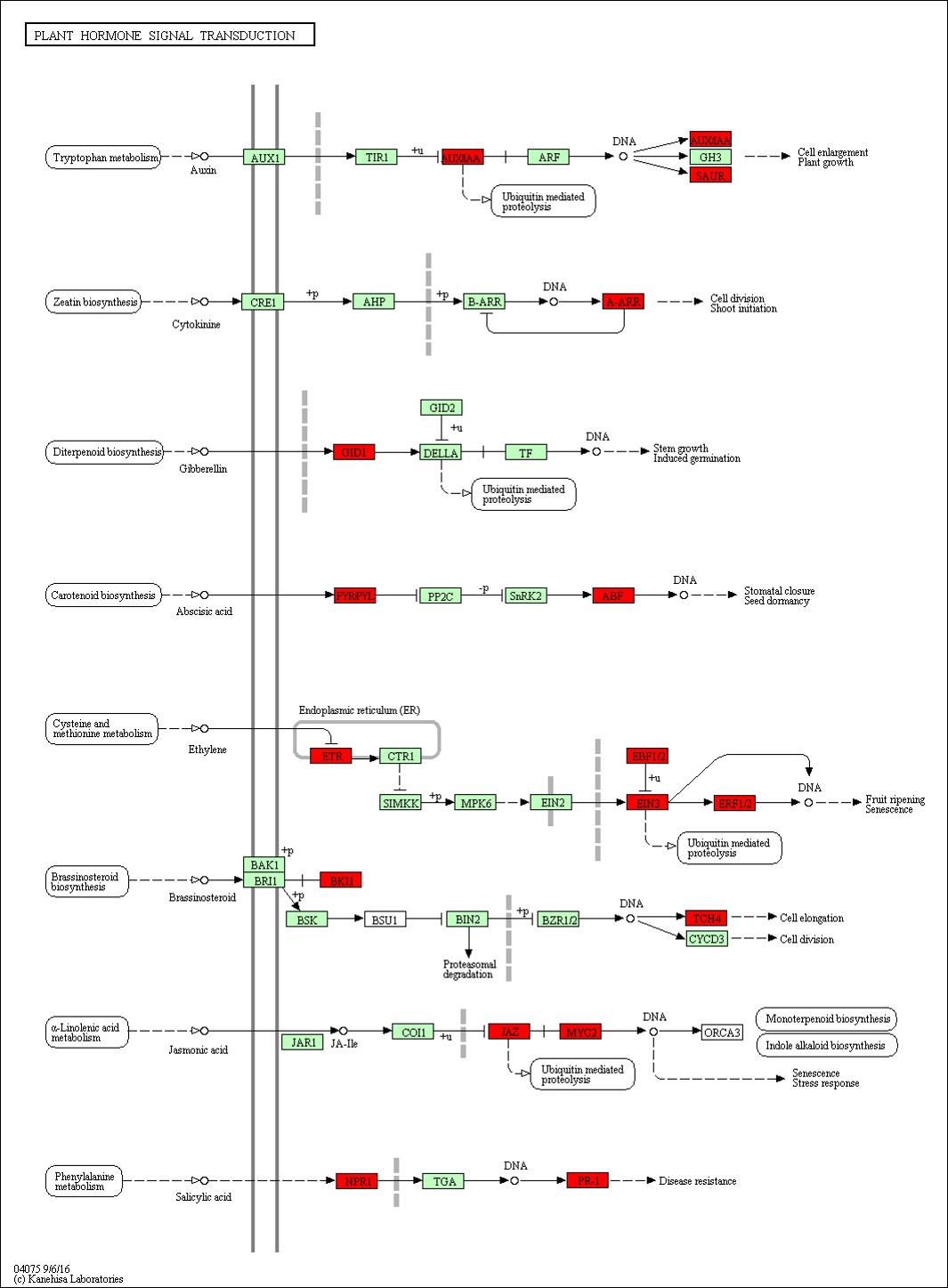

Supplement: Supplementary file 3 — Additional file 3: Figure S2. Schematic representation of plant hormone signal transduction taking place during the nematode resistance response based on the differentially expressed genes (up-regulated in PA99N82–4). All genes colored red are significantly up-regulated in PA99N82–4; green colored genes have been characterized for Solanum tuberosum in KEGG pathway. The image was generated using KEGG Mapper search and color pathway tool found at https://www.genome.jp/kegg/tool/map_pathway2.html. [file 12864_2019_6257_MOESM3_ESM.jpg]
